# Calorie restriction and stroke

**DOI:** 10.1186/2040-7378-3-8

**Published:** 2011-09-12

**Authors:** Silvia Manzanero, Mathias Gelderblom, Tim Magnus, Thiruma V Arumugam

**Affiliations:** 1 School of Biomedical Sciences, The University of Queensland, St Lucia, QLD 4072, Australia; 2 Department of Neurology, University Clinic Hamburg-Eppendorf, Martinistr, 52, D-20246 Hamburg, Germany

**Keywords:** calorie restriction, intermittent fasting, stroke, ischemia, brain

## Abstract

Stroke, a major cause of disability and mortality in the elderly, occurs when a cerebral blood vessel is occluded or ruptured, resulting in ischemic damage and death of brain cells. The injury mechanism involves metabolic and oxidative stress, excitotoxicity, apoptosis and inflammatory processes, including activation of glial cells and infiltration of leukocytes. In animal models, dietary energy restriction, by daily calorie reduction (CR) or intermittent fasting (IF), extends lifespan and decreases the development of age-related diseases. Dietary energy restriction may also benefit neurons, as suggested by experimental evidence showing that CR and IF protect neurons against degeneration in animal models. Recent findings by our group and others suggest the possibility that dietary energy restriction may protect against stroke induced brain injury, in part by inducing the expression of neurotrophic factors, such as brain-derived neurotrophic factor (BDNF) and basic fibroblast growth factor (bFGF); protein chaperones, including heat shock protein 70 (Hsp70) and glucose regulated protein 78 (GRP78); antioxidant enzymes, such as superoxide dismutases (SOD) and heme oxygenase-1 (HO-1), silent information regulator T1 (SIRT1), uncoupling proteins and anti-inflammatory cytokines. This article discusses the protective mechanisms activated by dietary energy restriction in ischemic stroke.

## Introduction

In the western world the average calorie intake has steadily risen as have associated diseases. Calorie restriction (CR) is defined as a decrease in energy intake without lowering nutritional value. This simple intervention has shown, in a wide range of laboratory animals, to extend lifespan and decrease the incidence of several age-related diseases [1]. In humans, CR can reduce markers of oxidative stress and inflammation [2,3], and can lower cardiovascular disease risk [4]. Dietary energy restriction also benefits neurons, as suggested by data showing that CR protects neurons against dysfunction and degeneration in animal models of epileptic seizure, stroke and neurodegenerative diseases [5,6].

The risk of ischemic stroke, the second major cause of morbidity and mortality worldwide, can be reduced through diet and lifestyle modification [7]. The mechanisms responsible for neuronal death caused by stroke are believed to involve metabolic compromise, over activation of glutamate receptors, cellular calcium overload, oxidative stress and inflammation [8]. Studies using *in vivo *and *in vitro *stroke models have identified several proteins and signalling pathways that can protect neurons against ischemic injury, including: neurotrophic factors, such as brain-derived neurotrophic factor (BDNF) and glial cell line-derived neurotrophic factor (GDNF); protein chaperones, including heat shock protein 70 (Hsp70) and glucose regulated protein 78 (GRP78); antioxidant enzymes, such as heme oxygenase-1 (HO-1) and the regulator of mitochondrial biogenesis PGC-1α. Several studies suggest CR may promote neuronal survival and plasticity in ischemic stroke, by inducing neuroprotective factors and suppressing inflammatory pathways. The present article reviews findings supporting the neuroprotective effects of CR and discusses the mechanisms activated by CR in ischemic stroke.

## Calorie Restriction

Experiments performed seven decades ago showed that CR increases the lifespan of rodents [9,10], and this has been widely replicated and extended, demonstrating an increase in both the mean and maximum lifespan of rats and mice maintained on CR [11-14]. More recently, it was shown that CR also slows aging in monkeys [15]. A number of physiological effects of CR that may contribute to its ability to increase lifespan have been documented in animal studies. Important among these are the preservation of metabolic functions despite aging [16], reduced body temperature and levels of oxidative stress [17,18], increased resistance to various types of stress [19], and enhanced immune function [20,21].

Another form of dietary stress studied alongside CR is intermittent fasting (IF). In rodents, it consists of alternating days of *ad libitum *feeding with days when only water is made available to the animals [22]. Human IF has involved alternating days eating less and more than the recommended daily energy intake [23]. CR and IF can improve risk factors for diabetes and cardiovascular disease in rodents [22-24], as well as delay, prevent or treat conditions responsible for mortality in rodents such as cancers and kidney disease [25-27]. When maintained on a CR or an IF diet, organisms ranging from yeast to monkeys exhibit increased resistance to many different types of stressors [19]. This is associated with increased resistance of cells in many different tissues to injury induced by oxidative, genotoxic and metabolic insults. The conservation of stress resistance responses to CR and IF across a range of species provides strong evidence that this mechanism contributes to the lifespan-extending action of dietary restriction.

## Cellular and molecular mechanisms underlying effects of calorie restriction on the brain

### Reduced oxidative damage

Mitochondrial ROS such as superoxide and peroxide anions, and their products, are a result of mitochondrial oxidative phosphorylation and cause oxidative damage to proteins, lipids and DNA. Accordingly, ageing is believed to be in large part due to cumulative damage caused by mitochondrial ROS [28], and an inverse correlation has been found between ROS production and longevity across mammalian species [29]. The brain is particularly susceptible to oxidative stress because of the high level of mitochondrial activity and the presence of heavy metal ions that can act as catalysts of oxidative reactions. Besides, the abundance of lipids in the nervous system makes them a prime target of oxidative damage. Hence, lipid peroxidation plays an important role in many neurodegenerative and psychiatric disorders [30]. Moreover, damaged molecules tend to accumulate in long-lived, post-mitotic neurons [31], making the situation worse and providing a connection between age and oxidative stress in the brain. In stroke, markers of oxidative damage to lipids and proteins have been found in animal models as well as in human patients, and levels of some of them correlate to stroke severity [32,33].

**Figure 1:**
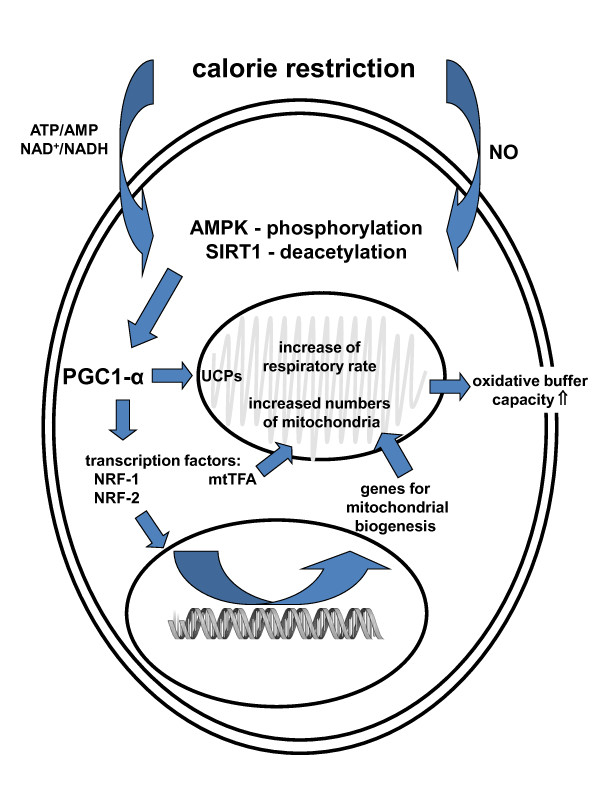
**Pathways of calorie restriction leading an increased metabolic rate**. Cells are sensitive to energy levels through molecules that detect ATP/AMP and NAD^+^/NADH ratios. Energy deficiency will activate the enzymes AMPK and SIRT1, which is also activated by NO, and through their respective phosphorylation and deacetylation, the activity of numerous substrates involved in metabolic functions will be modified. One of these is PGC-1α, which activates the transcription factors NRF-1 and -2 and mtTFA. The former two activate nuclear transcription of genes for mitochondrial biogenesis and the latter does the same in the mitochondria. This accounts for an increase in the numbers and size of mitochondria as well as an enhancement in the respiratory rate. All of these changes elevate the oxidative buffer capacity of the cell, augmenting its resistance to conditions of stress.

There is evidence that both CR and IF prevent oxidative damage by three major mechanisms: diminished production of mitochondrial reactive oxygen species (ROS), increased antioxidant defences and increased repair mechanisms for molecules that have been damaged as a result of oxidation [34]. Several studies have shown low levels of mitochondrial ROS generation in various tissues of CR rodents including the brain [35,36]. There is evidence that this is due to a mild enhancement of the mitochondrial respiratory rate, resulting in lower ROS release. Recent studies provided substantial evidence to confirm the link between respiratory rate, ROS release [37] and aging [38] by causing mild uncoupling in the passage of protons through the inner mitochondrial membrane from mitochondrial phosphorylation. This uncoupling is partly mediated by the so-called uncoupling proteins (UCP), whose levels are increased by CR in various tissues, including the neuron-specific UCP4 (Figure 1) [39]. There is evidence for the neuroprotective effects of UCP2, UCP4 and UCP5; however, their effects seem to encompass more than just mild uncoupling of the mitochondrial membrane and in some cases they appear to mediate protection through totally different mechanisms [40-43].

ROS scavengers, such as superoxide dismutase, glutathione peroxidase and catalase among others, are essential for antioxidant defence [44]. However, their levels or activity do not seem to be greatly affected by CR [45,46]. As for repair mechanisms for ROS-damaged molecules, it has been shown that CR reduces transcription levels of protein and DNA repair genes in skeletal muscle, but this seems to be partly a response to the lower damage caused by a lower metabolic rate [45]. An exception to this is the enzyme heme oxygenase-1 (HO-1), which is induced in various cell types by many stressful stimuli, including IF [47], and has anti-oxidant, anti-inflammatory and anti-apoptotic activities, which have been shown to contribute to mouse brain protection from focal ischemia [47]. Overall it seems, however, that the reduction of ROS produced in the mitochondria might be the most relevant mediator of CR-induced effects.

### Increased mitochondrial biogenesis

How CR decreases the metabolic rate in order to lower oxidative stress is not well understood. Moreover, this is in apparent contradiction with another CR effect, which is its proved ability to increase the number and promote the activity of mitochondria. A key mediator of these effects is the peroxisome proliferator-activated receptor γ (PPAR γ) coactivator 1α (PGC-1α), a protein central to mitochondrial biogenesis, whose activity explains the coordination of mitochondrial processes by environmental factors. PGC-1α is regulated through many mechanisms, of which AMP-activated protein kinase (AMPK) phosphorylation and silent information regulator T1 (SIRT1) deacetylation seem to be the most likely candidates to mediators of CR [48]. AMPK is activated by changes in the ATP/AMP ratio, whereas SIRT1 is activated by nicotinamide, which shows its dependence on NAD^+^/NADH balance. Both of these enzymes are, therefore, in tune with energy levels, showing them as prime targets for CR. In fact, CR has been shown to increase the activity of both AMPK and SIRT1, resulting in increased levels and activity of PGC-1α (Figure 1). SIRT1 is also activated by NO, another putative target of CR [49]. PGC-1α mediates processes relevant to mitochondrial biogenesis, including: i) up-regulation of transcription factors that activate transcription of mitochondrial genes in the nucleus, such as NRF-1 and NRF-2; ii) induction of transcription and replication of the mitochondrial genome, mediated by the mitochondrial transcription factor mtTFA, which is in turn activated by NRF-2. Besides, PGC-1α induces UCPs [50], which in turn result in lower ROS release. Therefore, CR does not seem to decrease the metabolic rate, as was formerly thought, but it appears to actually increase it [51], and this increase is responsible for its buffering effects on oxidative stress (Figure 1).

Corresponding to its beneficial effects on cell metabolism, PGC-1α deficiency is believed to mediate the neurodegenerative effects of AD, HD and PD, as decreased levels of this molecule were found in post-mortem analysis of patients [52-54]. Accordingly, PGC-1α and another member of the family, PGC-1β, have been reported to control mitochondrial density in neurons and reduce oxidative stress [55]. Despite the accumulating evidence of the effects of CR and PGC-1α on mitochondrial biogenesis, one study has claimed not to have found such increase, with levels of PGC-1α and essential mitochondrial proteins unchanged after 14 weeks of CR in rats [56]. These results only highlight how complex the mechanisms are that mediate CR effects and how far we are from elucidating how the process works.

### Increased cellular stress resistance

Another neuroprotective mechanism of CR is based on its putative effects as a mild stressor, activating cellular stress response pathways with upregulation of neurotrophic factors and heat-shock proteins, which in turn make the cells more resistant to neurodegeneration and ischemic insults. This is known as a preconditioning, or hormetic, effect, or the ability of a sublethal stressor to protect an organ from a subsequent lethal injury. The following molecules have been found to be up-regulated and mediate some of the effects of CR and IF in the mammalian brain.

#### Neurotrophic factors

Brain derived neurotrophic factor (BDNF) has been shown to be up-regulated by CR and IF in rodent and primate brain [47,57-59], whereas human evidence is still pending [60]. In a model of seizure, injection of BDNF blocking antibody reduced the positive effects of CR, demonstrating this molecule's essential role mediating the effects of CR in protection from excitotoxicity [57,58]. BDNF is a versatile neurotrophin with a central role in synaptic plasticity and consequently learning and memory. Its levels are very closely linked with diet, so that when food intake is high, and particularly if it is high in saturated fat, energy metabolism increases resulting in high production of ROS. Oxidative stress down-regulates BDNF, and thus negatively affects synaptic plasticity [61]. BDNF also has a function in turning down energy metabolism, so the effect is amplified. Conversely, CR or IF decrease oxidative stress, and this leads to the up-regulation of BDNF, resulting in increased synaptic plasticity. BDNF signals through the TrkB (tyrosine kinase) receptor, activating the Trk kinases which trigger a number of signalling pathways, including PLC (phospholipase C) γ, PI3K (Phosphatidylinositol 3Kinase) and the MAPK (mitogen activated protein kinase) pathway [62]. The resulting gene expression promotes synaptic plasticity, neurogenesis and cell survival. It is not surprising that BDNF is important in the protection from neurodegenerative diseases, such as PD and AD [63] and in the recovery from stroke (see below).

Glial cell line-derived neurotrophic factor (GDNF) has an important role in PD because it promotes survival of dopaminergic neurons whose malfunction accounts for the symptoms of the disease, and it also has a general role in the protection and development of many types of neurons (reviewed in [64]). Just as BDNF, GDNF signals through PLCγ, PI3K and MAPK pathways, and in fact these two neurotrophic factors seem to act in synergy, although it has been shown that BDNF has more powerful effects in the protection of dopaminergic neurons [65]. Neurotrophic factors and oxidative stress seem to be mutually negative regulators, because neurotrophins lower oxidative stress by up-regulating antioxidant proteins, providing an indirect role of CR on antioxidant functions. On the other side, oxidative stress down-regulates neurotrophins, providing a feedback loop that can be activated by high food consumption and is detrimental for the cell [66].

#### Heat-shock proteins

The main member of this family affected by CR is heat-shock protein (Hsp) 70. Levels of this chaperone in the rat brain have been shown to decrease with age up to 75% [67], and CR and IF can increase them [47,68]. Besides its role in protein folding, Hsp70 offers neuroprotection with its anti-apoptotic role downstream of cytochrome c and upstream of caspase-3 [69], and its role in the preservation of energy transfer in case of impaired mitochondrial metabolism [70]. Hsp70 is induced after a central nervous system insult, including seizure [71] and excitotoxic, oxidative and metabolic insults [68], which provides evidence for the role of this protein in the recovery from brain injury.

Glucose-regulated protein (GRP) 78 is a stress-induced chaperone in the endoplasmic reticulum, and as such it enhances the secretion rate and efficiency of specific proteins [72]. Besides, GRP78 promoter is enhanced in conditions of low glucose or oxygen, which are characteristic of ischemia and tumours. As a consequence of its function in the protection from protein misfolding, GRP78 has anti-apoptotic functions in neurons [73]. Like Hsp70, levels of GRP78 in brain have been seen to decrease with age [47], whereas they increase substantially under CR and IF conditions [47,68].

### Enhanced autophagy

Autophagy refers to the cellular process by which long-lived proteins and whole organelles get sequestered and degraded by lysosomes [74]. It is an essential process for the health of long-lived cells such as neurons, and, therefore, it is fundamental for the maintenance of the nervous system. Autophagy is heavily involved in synaptic growth and plasticity in *Drosophila *[75] and its disregulation has been linked to neurodegenerative diseases such as AD, PD and HD [76]. Autophagy is induced by oxidative stress via the activity of PI3K [77], and strongly inhibited by the mammalian target of rapamycin (mTOR, [78]). Even short term CR is known to enhance autophagy in neurons [79], and this is primarily mediated by inhibition of mTOR. Like PGC-1α, this molecule forms a nexus between diet and cellular changes, because of its ability to sense cellular ATP/AMP ratios, through its inhibition by AMPK, insulin and amino acid levels [80]. mTOR is down-regulated by CR and its inhibition plays an essential role in CR-mediated positive effects, including delayed aging, synaptic plasticity and delayed neurodegeneration, and most of these effects are mediated by its regulation of autophagy.

### Reduced inflammation

Inflammation, the complex, somehow nonspecific, process by which the body combats infection, is currently proving to have a dark side for it can have powerful negative effects in many non-infection mediated medical conditions. The presence of inflammation, even at low levels, can worsen the outcome of obesity, stroke, neurodegenerative and other diseases. Moreover, some chronic conditions arise as a result of unwanted inflammation, such as type II diabetes. Levels of inflammatory markers, such as C-reactive protein, tumor necrosis factor (TNF) and interleukin 6 (IL-6), increase with age and obesity, and decrease accordingly with CR [81], and the mediators of this decline are various, including the already mentioned Hsp70, PGC-1α and neurotrophic factors such as BDNF, but here we will focus on two key proteins involved which are SIRT1 and mTOR.

#### SIRT1

One of the substrates of the deacetylase SIRT1 is the nuclear factor κB (NFκB) subunit RelA which when deacetylated shows decreased ability to enhance transcription after TNF stimulation [82]. Because NFκB is the central transcription factor responsible for expression of many genes involved in inflammation, SIRT1 inhibits inflammation, and direct evidence of its activity has been shown in neuronal death by microglia inflammatory response to amyloid-β [83]. This immunoregulatory effect of SIRT1 adds to the list of advantageous effects of the enzyme which is strongly up-regulated by CR.

#### mTOR

The inhibition of mTOR has a dramatic effect in the suppression of inflammation, and in fact rapamycin, the drug from which it gets its name, has a strong immunosuppressive effect and it is currently used to minimize transplant rejection. This is due to the fact that mTOR, which is activated by the PI3K/Akt pathway, promotes cell growth and proliferation, cytokine production and signalling, all of which are essential for an efficient immune response [84].

## Molecular mechanisms of ischemic stroke induced brain injury

### Excitotoxicity

A significant proportion of ischemia-induced neuronal damage is mediated by toxic accumulation of excitatory amino acids. The lack of energy caused by the interruption of cerebral blood flow leads to failure of ion pumps, which results in inwards diffusion of calcium and sodium across the membrane along their concentration gradients, causing cellular swelling and depolarization [85]. Elevations of intracellular sodium become toxic and can contribute to necrotic neuronal death at early time points (minutes to hours) after ischemia. Elevations of calcium, however, activate ionotropic glutamate receptors. Glutamate, which is the major excitatory neurotransmitter in the brain, accumulates in the extracellular space and activates AMPA/kainate and NMDA receptors. Calcium ions enter the cell through these voltage-dependent and ligand-gated ion channels, resulting in the activation of a number of proteases, kinases, lipases and endonucleases, culminating in apoptosis [86,87]. It has been suggested that many neurons, particularly those in the ischemic penumbra, die by this mechanism involving glutamate-induced calcium influx [88].

### Oxidative damage

Neurons are normally exposed to baseline levels of oxidative stress, caused by free radicals from both exogenous and endogenous sources. These are highly reactive molecules with one or more unpaired electrons, which can react with DNA, proteins and lipids causing varying degrees of damage and dysfunction. Numerous experimental and clinical studies have documented increased levels of oxidative stress during all forms of stroke injury. Free radicals involved in stroke-induced brain injury include superoxide anion radical, hydroxyl radical and nitrous oxide (NO). Mitochondria are the primary source of ROS during ischemic or hemorrhagic stroke injury, which produce superoxide anion radicals during the electron transport process. Another potentially important source of superoxide in post-ischemic neurons is the metabolism of arachidonic acid through the cyclooxygenase and lipooxygenase pathways [89]. Following reperfusion in ischemic injury, ROS is also generated by activated microglia and infiltrating peripheral leukocytes via the nicotinamide adenine dinucleotide phosphate (NADPH) oxidase system [90]. NO is generated from L-arginine through one of several nitric oxide synthase (NOS) isoforms. The neuronal form of NOS, which requires calcium/calmodulin for activation, is produced by subpopulations of neurons throughout the brain [85]. Inducible NOS (iNOS) is produced by inflammatory cells, such as microglia and monocytes. These two isoforms are, for the most part, damaging to the brain under ischemic conditions, however a third isoform of NOS found in endothelial cells promotes vasodilation and may play a beneficial role following a stroke by enhancing reperfusion. NO diffuses freely across membranes and reacts with superoxide to produce peroxynitrite, another highly reactive free radical [91]. Both ROS and reactive nitrogen species are involved in activating several pathways involved in cell death following stroke, including apoptosis and inflammation. Lipid peroxidation also appears to play a prominent role in the pathogenesis of stroke. The mechanism whereby membrane lipid peroxidation induces neuronal apoptosis involves generation of an aldehyde called 4 hydroxynonenal, which covalently modifies membrane transporters such as Na^+ ^/K^+ ^ATPase, glucose transporter and glutamate transporter, thereby impairing their function [87,92].

### Inflammation

Besides its neurotoxic activity, calcium and free radicals can also activate inflammatory transcription factors, including NFκB [93]. These induce the expression of inflammatory cytokines (e.g. IL-1β, IL-6 and TNF), chemokines (e.g. monocyte chemotactic protein-1, MCP-1) and endothelial cell adhesion molecules (e.g. selectins and inter-cellular adhesion molecule 1, ICAM-1) among others [94]. There are several resident cell populations within brain tissue able to secrete pro-inflammatory mediators after an ischemic insult, including endothelial cells, astrocytes, microglia and neurons. Activated microglia produce several pro-inflammatory cytokines, as well as toxic metabolites and enzymes, and in addition, astrocytes play an important role in stroke-induced brain inflammation [94]. Because of the mixed nature of microglial and astrocyte products (both destructive and protective factors), the overall role of glial cells may differ at different time points following a stroke, with damaging effects occurring early (hours to days) and protective or regenerative activities occurring later (several days to weeks) [94].

## Calorie restriction and ischemic stroke

The first way in which CR protects from stroke is by preserving a healthy cardiovascular system. In a study of people who had been on a CR diet for an average of six years versus others on a typical American diet, it was found that CR reduced body fat, blood pressure and serum lipid and lipoprotein levels [95], which are well-known risk factors for ischemic stroke. The positive effects of CR and IF on systemic blood pressure have been extensively studied in animal models [reviewed in 4] and the mechanism seems to be mediated either by decreased activity of the sympathetic nervous system [96,97] or by modifications in activity of the hypothalamic-pituitary neuroendocrine pathways [98]. Age-related decrease in cerebral basal blood flow and brain vascular density was shown to be attenuated by CR in rats, and this appears to be mediated by alterations in growth hormone and insulin growth factor 1 (IGF-1) [99,100]. In addition, CR improves endothelial function and decreases circulating levels of inflammatory markers, both of which protect from atherosclerosis, a condition which is intimately linked to stroke [101].

However, once an ischemic insult has occurred, the rates of damage and recovery are affected significantly from CR at many different levels. This is because protective mechanisms are up-regulated by CR and those mechanisms that are down-regulated by CR are detrimental for stroke outcome. In this sense CR has a hormetic or preconditioning effect, which consists on subjecting the brain to small, harmless insults in order to induce tolerance to ischemia. CR restricts the number of nutrients that reach the brain cells and as a consequence switches on the defence machinery required to protect the cells from lack of nutrients. As a result, cells are now prepared to receive a more severe insult and as a consequence protected from it when it happens. CR pleiotropic effects protect the brain from ischemia by targeting excitotoxicity, oxidative damage, apoptosis, inflammation and autophagy.

### Excitotoxicity

**Figure 2:**
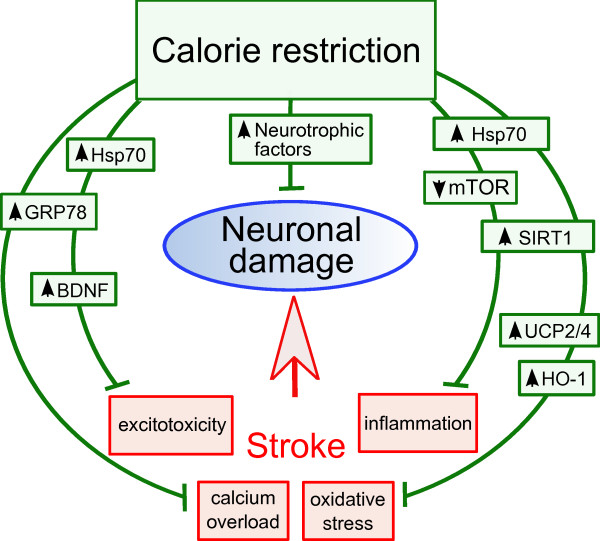
**Protective mechanisms of calorie restriction against neuronal cell death in stroke**. Stroke acts in detriment of neuronal health by different mechanisms, including exicitotoxicity, calcium overload, oxidative stress and inflammation, which can culminate in neuronal apoptosis. CR prepares neurons to bear each of these forms of stress by modifying the levels of key stress-response proteins. Certain proteins are up-regulated, such as the chaperones GRP78 and Hsp70, which protect from calcium overload and inflammation; neurotrophic factors such as BDNF, whose role is to protect the cells from excitotoxicity but are also key promoters of neurogenesis after stroke; SIRT1, a central mediator of many CR beneficial effects such as resistance to oxidative stress and moderation of inflammation (through its down-regulation of NFB); UCPs, which are thought to decrease the generation of ROS; and HO-1, with anti-oxidant properties. Hence, CR prepares brain cells at many levels to resist stroke-induced neuronal cell death and promote recovery after stroke.

CR has been shown to up-regulate mechanisms that protect the cells from glutamate excitotoxicity. At the tissue level, it increases the efficiency of astrocytes at taking up glutamate, reducing its availability and subsequent neuronal damage [102], which could be beneficial in the case of ischemia. Besides, once the neuron suffers from glutamate toxicity, all the pathways previously up-regulated by CR come to the rescue, improving the outcome by protecting neurons from cell death. In a mouse model of focal ischemia, mice subjected to IF displayed a smaller infarct volume, and this correlated with higher levels of BDNF, Hsp70, GRP78, HO-1 and other protective factors (Figure 2) [47]. The same is true for CR [103]. BDNF, which is up-regulated after ischemic stroke [104], has been shown to decrease the levels of extracellular glutamate in rat brain if applied 2 hours before an ischemic insult, and to counteract the tendency of inhibitory GABA receptors to reduce in number after ischemia, minimizing as a result the toxic effects of glutamate [105]. The chaperone Hsp70, which is also induced by ischemia [106], has a role in the protection of NMDA receptors and as such contributes to their normal function in toxic conditions. It also protects the presynaptic terminal by maintaining ion channel proteins in the presence of toxic levels of glutamate [107]. The endoplasmic reticulum associated GRP78, in turn, is responsible for maintaining the levels of intracellular calcium despite the insult, as hippocampal neurons treated with siRNA designed to inactivate GRP78 displayed higher levels of intracellular calcium upon glutamate treatment than control neurons, which is consistent with a role of calcium from the endoplasmic reticulum in excitotoxicity [108]. 2-deoxy-D-glucose, a molecule with similar neuroprotective effects as CR, increased the levels of GRP78, minimized the increase in intracellular calcium and protected from excitotoxicity [109]. Therefore, some of the proteins up-regulated by CR or IF can directly diminish the levels of glutamate excitotoxicity on neurons.

### Oxidative damage and apoptosis

As seen previously, the induction of protective mechanisms by CR accounts for lower basal levels of oxidative stress. However, these mechanisms must also be able to block the generation of ROS and minimize oxidative stress in ischemia to be useful in the protection of the brain from an ischemic insult. A central mediator of the protection from ROS in the brain is HO-1, which is induced by hypoxia, has direct antioxidant functions in the ischemic brain [110] and is up-regulated by IF (Figure 2) [47]. However, the lower oxidative damage encountered in neurons after stroke in CR animals, is not mainly caused by ROS scavenging but by a lower production of ROS. In this regard there is emerging evidence of the relevance of uncoupling proteins, as increased UCP2, 3, and brain-specific UCP4, gene transcription by CR have been shown in human skeletal muscle and brain respectively [39,111]. Overexpression of UCP2 in mice diminished neuronal damage after stroke by inhibiting generation of ROS and preventing apoptosis [40], while overexpression of UCP4 had similar effects in neurons subjected to toxins [39,112]. Besides, knock-down of UCP4 in primary hippocampal neurons increased mitochondrial calcium accumulation and cell death [39]. These effects were partly mediated by their prevention of ROS release, consequence of their uncoupling activity, but also by a more direct anti-apoptotic activity.

Both oxidative damage and apoptosis are direct consequences of the exicitotoxic stress imposed on neurons by ischemia, and neurotrophins [113], heat shock proteins [114], and other factors such as HO-1 [115] are known to protect cells from apoptosis. However, most of the current research interest is centred on SIRT1 as the central mediator of the defence against oxidative stress and apoptosis caused by ischemia. SIRT1 has been proposed to mediate neuron survival based on its broad deacetylase activity and has been linked to a positive stroke outcome. SIRT1 function as a deacetylase directly inhibits p53-dependent apoptosis in cortical neurons, and forkhead box (Fox) O3-dependent apoptosis in cerebellar granule neurons, when treated with DNA-damaging, apoptosis promoting agents [116-118]. Affecting either of these transcription factors has positive effects on stroke, as both p53 and FoxO3 have been found to promote cell death upon ischemic insult *in vitro *[119,120] and *in vivo *[121-123]. Another substrate of SIRT1 is the relA subunit of NFκB, which as a result fails to activate transcription of pro-apoptotic proteins in response to ischemia *in vivo *and *in vitro *[124]. SIRT1 also prevents apoptosis by activating the repair protein Ku70 following DNA damage. Under conditions of oxidative stress, Ku70 acetylates, which prevents its ability to sequester the proapoptotic factor Bax, but SIRT1 deacetylates Ku70, which allows it to bind Bax, disabling its apoptotic function [125,126]. This function of Ku70, whose levels increase after an ischemic insult, has been linked to a decrease in apoptosis after neonatal rat ischemia [127], and a Bax-inhibiting peptide, based on the Bax-Ku70 inhibiting domain, has proved to inhibit apoptosis and improve neurological outcome in rats subjected to global cerebral ischemia [128].

Another protein that prevents apoptosis in stroke, whose deacetylation is mediated by SIRT1, is PGC-1α. SIRT1 not only strongly activates PGC-1α by deacetylation but it also increases protein levels [129]. PGC-1α, which is also activated directly by oxidative stress, is responsible for protecting neurons from the excitotoxic effects of ischemia. PGC-1α has been shown to be induced after transient global ischemia, where it protects hippocampal neurons from delayed cell death, and knocking down the gene results in lower expression of UCP2 and the antioxidant enzyme superoxide dismutase 2 (SOD2) leading to neuronal death from oxidative stress [130]. Besides, there is *in vitro *evidence that an increase in PGC-1α in neurons subjected to oxygen and glucose deprivation results in the activation of NMDA receptors and inhibits the expression of p38 and ERK MAPK, protecting the neurons from death [131].

Surprisingly, despite its pleiotropic effects on the outcome of stroke, the effect of SIRT1 on recovery from an ischemic insult is not clear. There is evidence that SIRT1^+/- ^mice have a significantly worse outcome from focal ischemia [132], and drugs such as icariin and resveratrol that increase SIRT1 levels are protective [133,134]. However, another study reported the positive effects of nicotinamide, an inhibitor of SIRT1, on the outcome of brain ischemia, and the explanation given is that SIRT1 is a NAD^+^-dependent enzyme, and therefore in a moment of extreme nutrient deficiency such as stroke, the detrimental effects of its high energy consumption outweigh its aforementioned beneficial effects [135]. On another note, it has been reported that SIRT1's effects on neuroprotection might not be related to its deacetylase activity, as SIRT1's mutations lacking this activity continued to be protective in a model of low potassium-induced neuronal apoptosis [136]. Significant research effort is currently being applied to the understanding of the complexities of SIRT1 in the modulation of protection mechanisms following ischemia and the therapeutic possibilities of the enhancement of its activity.

### Inflammation and autophagy

The positive effects of CR on inflammation and its impact on stroke outcome are remarkable. On one side, having low basal levels of inflammatory cytokines in the circulation decreases susceptibility to stroke [137]. On the other side, CR and IF suppress the overproduction of inflammatory cytokines that is common after ischemic stroke (Figure 2) [47] and which is known to some extent to worsen stroke outcome [138]. SIRT1 and its inhibitory role on NFκB could have a lot to do with the lower transcription levels of inflammatory markers seen after stroke in CR organisms. However, another mediator is mTOR, a kinase which, as explained previously, has an important role in inhibiting autophagy and inflammation [74]. mTOR plays a role in cell growth, but it also has a function in post-mitotic cells such as neurons, and recently, evidence was shown for increased autophagy after only 48 hours of CR in cortical and Purkinje neurons [79], which correlated with decreased levels of mTOR. The function of autophagy in neurons is to dispose of toxins or damaged mitochondria and it is thought to play an important part in cellular detoxification in stroke. Supporting evidence for the role of autophagy in stroke comes from an *in vivo *model of transient focal ischemia, where autophagy was detected one day after the insult and then decreased over a period of six days [139]. However, evidence also suggests that autophagy could lead to neuronal death, promoting the assumption that autophagy is a tightly regulated balance that can go one way or another [140,141]. Another consequence of mTOR inhibition, as shown by treatment of cultured brain slices with its inhibitor rapamycin, is the suppression of the detrimental post-ischemic long-term potential, but without affecting synaptic plasticity [142], which would otherwise lead to apoptosis. mTOR inhibition also contributes to a positive stroke outcome by decreasing inflammation and immune system activation (Figure 2) [143]. This is evident even in microglia, where mTOR has an effect on activation by hypoxia which is downstream from iNOS and forms part of the PI3K/Akt pathway. This effect, in the case of ischemia, could be responsible for the release of inflammatory molecules by microglia with neuronal death as a result of this inflammation [144].

### Other effects of CR in stroke

#### Neurogenesis and angiogenesis

These two processes are essential for the reconstruction of brain tissue after stroke, which requires the generation of new neurons and neuronal connections as well as the irrigation of these neurons. The primary mediators of ischemic tissue recovery after stroke are BDNF and vascular endothelial growth factor (VEGF). It has been shown that the rate of neuronal production is enhanced after stroke and traumatic brain injury (reviewed in [145]), and BDNF, the mediator of neurogenesis in rodent models of stroke, is upregulated by CR [146]. Moreover, it has been shown that 25% CR for three months accounts for increased circulating levels of BDNF in obese humans. VEGF, like other angiogenic factors, is also essential for the recovery of brain tissue, as blood vessel formation has important functions in revascularization of the tissue as well as secretion of growth factors and chemokines which support the survival of new neurons. VEGF expression, enhanced by the hypoxia induced factor 1 alpha (HIF-1α) increases with ischemia and contributes to neuroprotection, neurogenesis and angiogenesis [147], as well as blood brain barrier protection. Besides, VEGF is upregulated by CR mimetic resveratrol, which also upregulates other important angiogenic protein, matrix metalloproteinase 2 (MMP-2) [148]. Together they contribute to blood vessel formation in the post-ischemic tissue. Another mediator of revascularization enhanced by CR is adiponectin, which upon ischemic insult increases angiogenesis mediated by activation of AMPK and eNOS, as has been observed in hindlimb ischemia [149]. Adiponectin, a metabolic modulator produced in adipose tissue whose circulating levels are increased in CR and IF [150,151], has also been found to have a positive effect in the recovery from brain ischemia [152].

#### Regulation of circulating stress hormones

One of the ways in which CR and IF have been shown to improve the outcome of stroke is through endocrine regulation. Adrenocorticotropic hormone (ACTH) shows an interesting pattern in rats: basal levels are higher under IF, but under stress conditions the increase is smaller than in control animals, suggesting an improved response to stress [98]. Since levels of ACTH and the hormone it induces, cortisol, increase dramatically after stroke and have been correlated with lesion size and neurological deficit in human patients [153,154], smaller increases of these hormones after stroke would seem to be beneficial. It has also been shown that CR down-regulates somatotropic signalling in mice [155], and that mice deficient in growth hormone show an increase in life span, just like those subjected to CR. Moreover, mice deficient in growth hormone that undergo CR do not show any further extension in life span, suggesting a partial dependency between both mechanisms which is mediated by insulin-like growth factor I and insulin [156].

## Age modifies cell stress pathways and stroke outcome

**Figure 3:**
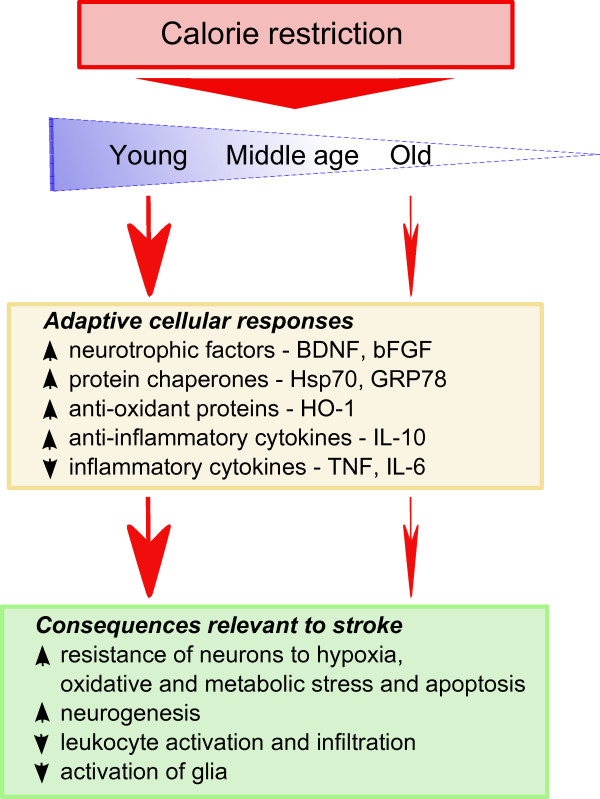
**The protective effect of calorie restriction on stroke diminishes with age**. In a recent work by our group [47], mice from different age groups were subjected to intermittent fasting for the same time period before suffering ischemic stroke. The adaptive cellular responses, in the form of neurotrophic factors, protein chaperones, anti-oxidant and anti-inflammatory environments, were strongly favoured in young mice. However, aged animals didn't show such a beneficial response. As a result, IF young mice were protected from stroke but the extent of the protection was weak or null in aged mice. These results show that the protective effects of IF against stroke are age-dependent.

Because aging is a major risk factor for stroke, and stroke outcome is poorer in the elderly, our group recently tested the hypothesis that aging impairs the ability of brain cells to respond adaptively to IF and so to survive a stroke after this regimen. Our findings suggested that aging compromises the ability of energy restriction to protect the brain against ischemic injury and improve functional outcome in stroke. The neuroprotective effect of IF was robust in young mice, was diminished in middle-aged mice, and was lacking in old mice (Figure 3) [47]. Our analysis of neurotrophic factors, stress resistance proteins, and cytokines suggests mechanisms by which aging impairs the ability of IF to protect brain cells against a stroke. Levels of BDNF and basic fibroblast growth factor (bFGF) were diminished in the cortex and striatum of old mice compared with young mice. The amounts of BDNF and bFGF were increased by IF to much higher levels in young compared with middle-aged and old mice [47]. Furthermore, levels of cellular stress protection proteins examined (Hsp70, GRP78, and HO-1) were elevated in response to IF and stroke in young mice, but with greatly diminished responses in middle-aged and old mice [47]. In addition, proinflammatory cytokines TNF and IL-6 levels increased during aging, and decreased in response to IF, particularly in young and middle-aged mice [47]. These findings suggest that reduction in dietary energy intake differentially modulates neurotrophic and inflammatory pathways to protect neurons against ischemic injury and these beneficial effects of IF are compromised during aging (Figure 3).

## Concluding Remarks

The results reported in this review provide evidence to confirm CR as an easy, cost-effective and efficient measure not only for the prevention of stroke, but also for the reduction of damage should stroke occur. On the other side, there is little evidence on the efficacy of CR as a treatment for stroke. One study reported the neuroprotective benefits of CR after traumatic brain injury in rats, showing that, in this context, 24 hours of fasting after a moderate injury resulted in lower oxidative stress and calcium influx, and improved mitochondrial function [157]. Experiments by another group on rat myocardial ischemia followed by IF revealed improved heart function and angiogenesis, and lower apoptotic rates in the IF rats compared to the control group. These effects were mediated, among others, by BDNF and VEGF [158]. A similar, positive result was encountered in rats subjected to spinal cord injury, where those that underwent IF after the procedure showed improved plasticity and recovery of neurons, accompanied by a 2 to 6-fold increase in TrkB, the BDNF receptor [159]. These results contrast with those obtained in a gerbil model of global ischemia (5 minutes followed by reperfusion), in which no improvement was found in animals subjected to CR after the intervention [160]. These studies, however, are promising enough to warrant additional research to find out how best, and to what extent, CR can help recover from stroke after the injury has occurred.

## Competing interests

The authors declare that they have no competing interests.

## Authors' contributions

SM has been involved in literature search and writing as well as drafting the manuscript. TM has been involved in drafting the manuscript. MG has been involved in drafting the manuscript. TVA has been involved in literature search and writing as well as drafting the manuscript and has given final approval. All authors read and approved the final manuscript.
